# Integrating Patient Reported Outcome Measures (PROMs) into routine nurse-led primary care for patients with multimorbidity: a feasibility and acceptability study

**DOI:** 10.1186/s12955-021-01748-2

**Published:** 2021-04-26

**Authors:** Ian Porter, Antoinette Davey, Jaheeda Gangannagaripalli, Jonathan Evans, Charlotte Bramwell, Philip Evans, Chris Gibbons, Jose M. Valderas

**Affiliations:** 1 Health Services and Policy Research, Exeter Collaboration for Academic Primary Care (APEx), University of Exeter, Magdalen Campus, Smeall Building, Room JS02, Exeter, EX1 2LU UK; 2 University of Strathclyde Institute of Pharmacy and Biomedical Sciences, Glasgow, UK; 3 NIHR Clinical Research Network, England, UK; 4 Division of Internal Medicine, Department of Symptom Research, The University of Texas MD Anderson Cancer Center, Houston, TX USA; 5 St Leonard’s Practice, Exeter, UK; 6 NIHR PenARC, University of Exeter, Exeter, UK

**Keywords:** Primary health care, Multimorbidity, Patient-Reported Outcome Measures (PROMs)

## Abstract

**Background:**

The use of Patient Reported Outcome Measures (PROMS) in clinical practice has the potential to promote patient-centred care and improve patients’ quality of life. Individualized PROMs may be particularly helpful in identifying, prioritizing and monitoring health problems of patients with multimorbidity. We aimed to develop an intervention centred around PROMs feedback as part of Primary Care annual reviews for patients with multimorbidity and evaluate its feasibility and acceptability.

**Methods:**

We developed a nurse-oriented intervention including (a) training of nurses on PROMs; (b) administration to patients with multimorbidity of individualized and standardized PROMS; and (c) feedback to both patients and nurses of PROMs scores and interpretation guidance. We then tailored the intervention to patients with two or more highly prevalent conditions (asthma, COPD, diabetes, heart failure, depression, and hip/knee osteoarthritis) and designed a non-controlled feasibility and acceptability evaluation in a convenience sample of primary care practices (5). PROMs were administered and scores fed back immediately ahead of scheduled annual reviews with nurses. Patients and nurses rated the acceptability of the intervention using with a brief survey including optional free comments. Thematic analysis of qualitative interviews with a sample of participating patients (10) and nurses (4) and of survey free comments was conducted for further in-depth evaluation of acceptability. Feasibility was estimated based on rates of participation and completion.

**Results:**

Out of 68 recruited patients (mean age 70; 47% female), 68 completed the PROMs (100%), received feedback (100%) and confirmed nurse awareness of their scores (100%). Most patients (83%) “agreed”/”strongly agreed” that the PROMs feedback had been useful, a view supported by nurses in 89% of reviews. Thematic analysis of rich qualitative data on PROMS administration, feedback and role in annual reviews indicated that both patients and nurses perceived the intervention as acceptable and promising, emphasizing its comprehensiveness and patient-centredness.

**Conclusions:**

We have developed and tested an intervention focusing on routine PROM assessment of patients with multimorbidity in Primary Care. Preliminary findings support its feasibility and a high degree of acceptability from both patients and nurses. The next step is to conduct a full-scale trial for evaluating the effectiveness of the proposed intervention.

## Background

Patient Reported Outcome Measures (PROMs) elicit measurements of any aspects of health status directly from patients themselves [1]. The use of PROMs in health care is a key approach for operationalizing patient-centred care [2], a core value of health systems. Conceptual dimensions of patient-centredness, as identified in a literature review conducted by Mead and Bower [3], include a biopsychological perspective and seeing the patient-as-a-person. PROMs address these dimensions by being responsive to the needs and preferences of individuals by incorporating social and psychological dimensions of health facilitating understanding of the individual’s experience of illness. Information on the outcome of care as perceived by patients themselves is essential for ensuring that the delivery and evaluation of care is respectful of and responsive to individual patient preferences, needs, and values. PROMs have traditionally been used most frequently in clinical settings for research purposes and the evaluation of the effectiveness of health interventions [4, 5]. They have been used more recently as part of quality improvement initiatives using generic and intervention specific PROMs, such as the NHS England PROMs programme [6] or the International Consortium for Health Outcomes Measurement (ICHOM) [7], both of which focus on evaluations at the health care provider level; or the Patient-Reported Indicator Surveys (PaRIS) initiative of the Organisation for Economic Co-operation and Development  (OECD), which aims to elicit measurements at a country level [8]. There is increasing recognition, however, of their potential use in routine clinical care [4, 5, 9] for supporting clinical decision making, monitoring of disease progression and response to treatment, eliciting patient preferences for specific outcomes, and aligning patients and professionals evaluation of health problems [5, 10]. The evidence is still not conclusive and additional research is needed for the identification of the most promising approaches to PROMs measurement and feedback [11].

The potential of using PROMs for supporting clinical management of patients presenting with more than one health condition, a circumstance defined as multimorbidity, is of particular interest [12] but has been not yet been explored. People with multimorbidity are increasingly the norm in Primary Care, who tend to be older than other patient groups [13], have increased use of healthcare services compared with other members of the population, yet face worse health outcomes [13, 14].

Current models of care for long term conditions in England, Wales and Northern Ireland rely on annual reviews. The purpose of such annual reviews is to ensure patients are taking the right medication and receiving the best possible care. Typically, there are separate reviews for each condition (frequently conducted by nurses) with little, if any, explicit integration of management across conditions [15, 16]. A variety of complex interventions have been developed in recent years to address this gap, but the evidence for their effectiveness is sparse [17]. Current guidance for the clinical management of patients with multimorbidity proposes that in the current circumstances the focus of care should be on improving the quality of life of patients in a way that is responsive to their individual needs, preferences for treatments, health priorities, lifestyle and goals [18]. Routine monitoring of patient-reported outcomes with PROMs would thereby facilitate the consistent evaluation of this aim.

Success in implementing PROMs in clinical practice requires careful coordination and planning [19, 20]. A framework for the implementation of PROMs in clinical practice has been recently proposed based on existing research and implementation of evidence [5]. It considers the use of condition specific and generic standardized PROMs along with individualized measures, which explicitly focus evaluations on those areas that are most relevant to each patient, as identified by patients themselves [1]. The framework, consistent with the biopsychosocial model [21], integrates monitoring of disease specific patient-centred outcomes with continuous evaluation of patient priorities and goals [5, 10, 20, 22] and proposes that feedback of PROMs results to patients would enhance their health care activation, a key component and determinant of success in the Chronic Care Model [12, 23, 24]. Findings of a subsequent realist synthesis on PROMs feedback in health care supports this perspective [2, 20, 25]. A PROMs based Primary Care review for patients with multimorbidity could potentially meet the objectives and processes of care proposed in current clinical guidance.

## Objectives

(a) To develop an intervention using real-time administration and feedback of PROMs for Primary Care patients with multimorbidity; (b) to assess the feasibility of its implementation in clinical practice; (c) to gain insight into it acceptability, and potential risks and benefits.

## Methods

### Development of intervention

We developed a nurse-oriented PROMs based patient-centred intervention aimed at people with multimorbidity in general practice focusing on goal setting, prioritization and monitoring. We followed Medical Research Council (MRC) guidance [26] in that the intervention was informed by theory [5, 27, 28] and evidence [29–32], the individual components were clearly described and were reproducible, and feasibility work was carried out to determine if the intervention could be implemented as intended, without undue risk, and if it was acceptable to patients and nurses.

**Fig. 1:**
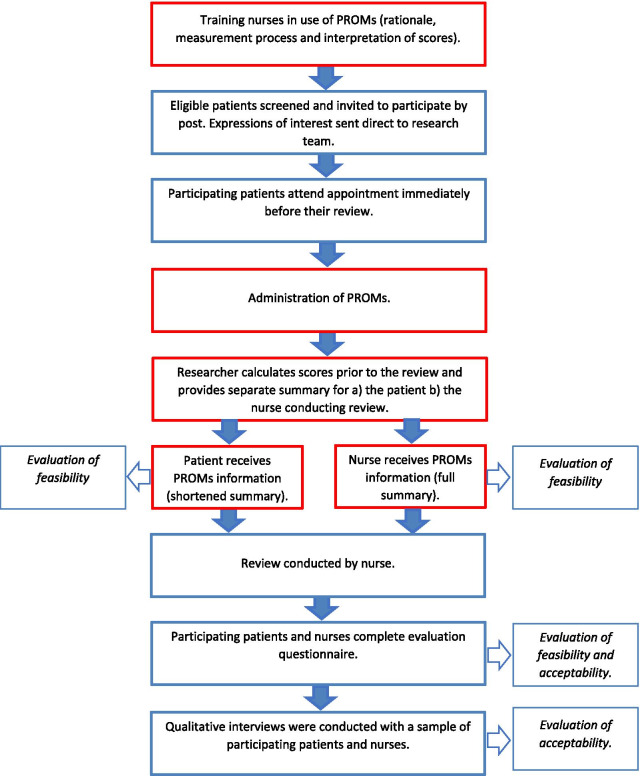
Flowchart showing key stages in study design (intervention components in red)

Following a review of theory and evidence as well a large number of previous interventions [11], we designed a nurse-oriented intervention for primary care patients with multimorbidity (Fig. 1) which included the following components:

1. *Nurse training* Face to face training of nurses on the use of PROMs delivered by members of the research team in a one-hour interactive workshop format covering the following elements:
  a A general presentation explaining patient-reported outcome measures, their different uses in clinical practice, and positive outcomes derived from their use.
  b Explaining the characteristics of the PROMs that will be used in the intervention, including how to interpret the results.
  c An open floor to discuss any questions the nurses might have.

2. *PROMs administration*: Administration of three types of measures to Primary care patients with multiple long-term conditions immediately before combined annual reviews for all their relevant conditions:
  a A set of condition specific PROMs (at least one for each relevant condition)
  b A generic, standardized PROM (EuroQol EQ5D) [33] and
  c An individualized PROM (Patient Generated Index (PGI) [34].
3. *PROMs feedback* Provision to patients and their nurse of real-time structured feedback of PROMs scores and interpretation guidelines using personalized forms:
  a Patient feedback: a simple summary of the results (individual scores for each standardized instrument and list of health priorities from the individualized instrument)
  b Nurse feed-back: including the previous information as well as:
    i Interpretation of scores: direction and range of theoretical scores; mean scores in reference samples; and
    ii Recommendations for the management of each clinical condition based on PROMs results based on best available clinical guidance
4. *Annual review* A nurse-led person-centred annual review of all the relevant chronic conditions as informed by PROMs results.

### Evaluation of the feasibility and acceptability of the intervention

We designed a non-controlled intervention study to test the feasibility and acceptability of the intervention and to gather information for its further refinement. It was beyond the scope of this study to conduct a controlled evaluation of the effectiveness of the intervention.

For the feasibility study, the intervention was tailored specifically to patients with any combination of the following conditions: asthma, COPD, diabetes, heart failure, depression, and osteoarthritis of either the hip or the knee. We selected these highly prevalent conditions because of their different impacts on symptoms and functioning in the physical, mental and social domains of health, and distinct patterns of clinical progression.

Instruments were selected based on previous reviews of available instruments for measuring PROMs for these conditions [35–40]. Each of these selected instruments are well established, valid, reliable and widely used measures. In addition, for asthma and COPD additional measures included in the Quality and Outcomes Framework, a quality improvement initiative widely endorsed by Primary Care practices in the UK, were selected (Table 1).

**Table 1:** Patient-reported outcome measures used in intervention

Generic	EuroQol (EQ-5D-5L) [49]
Asthma specific	Mini Quality of Life Questionnaire (mini-AQLQ) [50] Royal College of Physicians (RCP) 3 asthma questions [51]*
COPD specific	Clinical COPD Questionnaire (CCQ) [52] MRC breathlessness scale [53]*
Depression specific	Patient Health Questionnaire (PHQ-9) [54]
Diabetes specific	Diabetes Health Profile (DHP) [55]
Heart failure specific	Minnesota Living with Heart Failure Questionnaire (MLHFQ) [56]
Osteoarthritis specific	Oxford Hip Score (OHS) [57] Oxford Knee Score (OKS) [58]
Individualized	Patient Generated Index (PGI) [34]

^*^Included in the Quality and Outcomes Framework

Training, as well as patient and nurses’ feedback forms, were tailored accordingly (Appendix 1 and 2). The information was systematically retrieved from several different sources. Direction and range of scores were taken from the relevant manuals and original papers. Scores in relevant Primary Care UK samples were obtained from bespoke systematic searches of the literature in PubMed, ISI Web of Science and Google Scholar for each instrument [29]. Recommendation for management of the clinical conditions utilizing PROMs scores was based on a systematic review of clinical practice guidelines and quality indicators in reference sources for clinical care in the UK. The National Institute for Health and Care Excellence (NICE) produces and disseminates clinical practice guidelines for the chronic diseases and hosts a repository of indicators of quality of care for the management of chronic conditions [30, 41].

The involvement of patients was vital to the study design. A group of people diagnosed with chronic conditions was specifically  convened and supported by the Patient and Public Involvement team of the National Institute for Health Research Applied Research Collaboration South West Peninsula (NIHR PenARC). Feedback from the group helped to shape PROMs administration booklets, information sheets, and letters of invitation.

Five Primary Care practices in the South West of England were recruited between July 2015 and February 2016. This convenience sample, which enabled us to draw on practices both easily accessible and willing to participate in the study [42], aimed to maximize variation in terms of size and urban vs. rural locations. Our target was to recruit around 15 patients per practice, and 75 patients in total, who were due to attend a review, and to recruit all nurses conducting the reviews for specific PROMs training (outlined previously). No formal sample size calculation was performed. Given the variation in practice size, we wanted to establish if this target was feasible, especially in smaller, rural practices.

Searches of electronic records using Read codes identified eligible patients diagnosed with any combination of the proposed conditions and who were due for a review for any of those conditions in the subsequent six months. Exclusion criteria included inability to communicate in English and being under 18. A practice physician screened the list of eligible patients to assess suitability. Subsequently, patients were invited by mail to participate. Interested participants contacted the research team directly, who arranged to meet them at their practice immediately before their review.

After providing informed consent participating patients completed generic, condition specific and individualized PROMs in a personalized booklet, with assistance from the researcher if required, immediately ahead of scheduled annual reviews using paper forms. Personalized PROMs summaries were immediately provided to patients and nurses so that they could inform the annual review.

We evaluated feasibility by establishing: (1) the proportion of participants who completed the PROMs, including the individualized instrument; (2) the proportion of patients who completed the PROMS who received personalized feedback ahead of their review; and (3) the proportion of reviews for which the patient confirmed their nurse had received the personalized feedback ahead of their review.

We used mixed methods for evaluating the acceptability of the intervention. The combination of qualitative and quantitative viewpoints, data collection, and analysis enabled us to provide a balanced evaluation of the study. Data were collected in two stages: immediately after the review via brief surveys of participants and at a later date via qualitative interviews. After completing the PROMs review patients and nurses completed a feedback survey incorporating both standardized questions on the acceptability of the information and open-ended questions (Appendix 3 and Appendix 4). Data for Likert scale items were analysed using descriptive statistics for ordinal data, and open-ended questions were thematically grouped and synthesized. Ten patients and four nurses participated in face-to-face semi-structured interviews exploring the acceptability of the intervention, along with potential barriers and facilitators and risks associated with its implementation. Interviews were audio-recorded and typically took place one to two months after the original PROMs review took place. Qualitative analysis of verbatim transcriptions was conducted by two researchers according to the following steps: familiarization; agreement on thematic coding structure and descriptive labels; thematic coding with NVivo 11 [43]; production of NVivo reports for each theme; identification of line of argument in each report with One Sheet of Paper (OSOP) method [44]; and critical reassessment, including identification of negative cases or outliers, for example, any viewpoints at odds with the majority of the data which were nevertheless important to capture.

## Results

### Evaluation of the feasibility of the intervention

Training was successfully provided to all the 12 participating nurses (100%). Out of 223 patients subsequently screened as eligible by the practices and invited by them via post, 75 expressed an interest in taking part by replying to the research team (33.6%). Arrangements were made with 68 patients (90.1%) for their participation while it was not possible to make further contact with the remaining seven. Participants were aged between 48 and 90, with a mean age of 70; and 47% were women. Forty-seven participants (69.1%) were diagnosed with 2 (out of 6) eligible conditions, 14 participants (20.6%) with three, and seven (10.3%) with four. The most common combinations of conditions were diabetes and osteoarthritis (13%), diabetes and COPD (12%), and diabetes and heart failure (7%).

All 68 patients (100%) attended the practice on the day of the review, all of them (100%) completed the relevant PROMs and all of them (100%) received personalized feedback. Patients also confirmed that the nurse was aware of their scores in all the consultations (100%). All nurses (100%) also received the personalized feedback, along with additional information including the raw scores and how to interpret them.

PROMs scores for standardized measures were similar to available published scores in Primary Care UK samples (Table 2). Common health priorities identified by participants in their responses on the individualized PGI included aspects of functioning such as *mobility*, *walking* and *exercise*. A number of psychosocial priorities were also identified encompassing aspects such as mental health, social life and playing with their grandchildren. Sensitive health issues, for example, erectile dysfunction were also highlighted.

**Table 2:** Participants Patient Reported Outcome Measures (PROMs) scores

Patient reported outcome measure (PROM)		Theoretical range (worst-best)	Observed range	Observed mean	Reference mean*
Patient Generated Index		0–100	1–9	3.89	N/A
Euro-QoL-5D VAS		0–100	20–100	67.0	69.8
Asthma Mini Quality of Life Questionnaire		7–1	3–6.6	5.2	5.5
Clinical COPD Questionnaire		0–6	0–4.5	1.7	2.6
Oxford Knee Score		0–48	4–46	27.5	Pre-op: 19.4 Post-op: 36.1
Oxford Hip Score		0–48	11–48	30.9	Pre-op: 18.2 Post-op: 40.1
Minnesota Living with Heart Failure Questionnaire		0–105	1–68	30.7	46.6
Patient Health Questionnaire		27–0	15–1	8.9	15.5
Diabetes Health Profile	Psychological distress	0–44.4	0–100	7.5	20.1
	Barriers to activity	0–71.5	0–100	12	23.9
	Disinhibited eating	0–93.3	0–100	27.4	34.6

*Mean values in Primary Care samples obtained from the literature

### Evaluation of the acceptability of the intervention

Nurses and patients completed evaluation questionnaires for 62 and 60 respectively of the 68 completed reviews (Appendix 5 and 6). 93% of patient respondents would not have preferred more detailed information and 92% “agreed”/”strongly agreed” that the information was easy to understand. Nurses “agreed”/”strongly agreed” that the information was easy to understand in 95% of visits of completed exit questionnaires. 90% of patient  responses found the intervention helpful in prioritizing their health issues and 83% found the information helpful for the subsequent review (89% for nurses). 76% “agreed”/”strongly agreed” that they would like such information to be routinely included as part of their care.

Thematic analysis of the semi-structured one to one interviews (Tables 3 and 4) was supplemented by feedback in the open-ended responses elicited in the post-review evaluation questionnaires (Appendices 5 and 6).

**Table 3:** Thematic analysis of patient interviews

Theme	Subcategories	Description	Significant statement examples
Patient experiences patients completing PROMs	Ease	Ease of use of patients completing PROMs	“Everything seemed easy, there is nothing difficult about it at all.” (1008) “They were asked in a way that was easy to understand and accept.” (1009)
	Challenge	Challenge involved in completing PROMs	“It was really interesting to do. I enjoyed the challenge of finding the answers.” (1009)
	Comprehensiveness	Comprehensiveness of the PROMs used in the intervention	“I think it's you, know quite comprehensive.” (2007) “I came away from the interview thinking you’d covered most bases. I couldn’t think oh why didn’t he ask that or why didn’t he measure that.” (2017)
	Electronic administration	Views on the desirability of electronic administration	“Don't bother me either way.” (4009) “The personal contact in my view is essential.” (3009)
PROMs feedback	Detail	Views on level of detail required (simplicity versus inclusion of supporting information)	“The simplicity of it is the benefit really.” (4007) “It was absolutely brilliant’… ‘that is the benefit of it, it’s simple and anyone can look at it… you don’t need to put three charts together you have a very clear picture.” (1009) “I think if it had a few more clinical bits on it, and I’m talking about very short clinical bits on there… such as the results of their last blood test, or whatever it is pertaining to their particular health conditions, then I think that would be a really good document.” (5003)
	Awareness	Extent to which the PROMs summary encourages patient awareness of their health	“…it just puts down in black and white how I felt at the time.” (5004) “It was good, it’s highlighted the fact that I’ve been lucky I think since my knee’s been done that it’s had a good effect on the diabetes and makes me feel lucky as I say with both problems that I’ve had.” (4007) “I came back and discussed it with my husband and we talked about some of the things that had been raised… we then discussed some of the aspects of my condition that I perhaps hadn’t considered before so that for me was really good”. (5003)
	Prioritization	Extent to which the PROMs summary encourages patient prioritization	“It’s all too easy to assume that everyone needs what we think they need, or have problems with what we think they have problems with, and I guess if someone looked at me from the outside and saw me wobbling along they would probably think that my mobility problems were the worst part but it’s actually the pain and lack of sleep, that’s the bit that makes it hardest. So again that’s changed the priority from the outside view to my view.” (1009) “It brings your attention to what your priorities are.” (3008)
PROMs as part of routine care	Patient monitoring	Extent to which the intervention encourages patient monitoring	“I think looking at it may be used in the future it really does make it feel like a tool which can be, should be, ought to be used.” (1009) “Yeah, I think it’s a good thing. From my point of view it’s either going to highlight a problem or give you a reassurance that you know things are remaining good.” (4007) “I think that was good because I think in two years’ time you could repeat it, the whole questionnaire and you’d possible come out with different results.” (1009)
	Barriers	Barriers to implementation (such as resource pressures)	“I wonder what use it’ll be at the end of the day to GPs and that because they’re all so busy and they’ve got so much else on their plates… whether they worry about stuff like this I don’t know… if they just brush it under the carpet who knows… so many things are changing down our local GP practice that you don’t know where they are half the time.” (1008)

**Table 4:** Thematic analysis of nurse interviews

Theme	Subcategories	Description	Significant statement examples
PROMs feedback	Detail	Views on level of detail required (simplicity versus inclusion of supporting information)	“I found it very simple to look at and it’s very quick to get that information within a few seconds so that’s good when time is at a premium.” (3001p) “It was great. Nice and simple. I like simple…” (5001p) “I like the way that was laid out, that was good and clear and the actual sort of priorities written on the front.” (4001p) Supporting information: “I did refer to that because it was helpful just to understand a little bit more about where the patient had graded themselves.” (3001p) Supporting information: “For us to take this information further it was useful to have the background as well.” (3001p)
	Awareness	Extent to which the PROMs summary encourages patient awareness of their health	“I think that’s a really good idea, absolutely. And it doesn’t have to be just for chronic disease either, a patient very often comes in here with more than one issue, so if they’d had a chance to reflect on it and let us know about it in advance we might have more chance to prepare for them coming in the first place.” (5001p)
	Prioritization	Extent to which the PROMs summary encourages patient prioritization	“…making sure that we’re not missing the thing the person came into talk about.” (4001p) “I used their health priorities on your form as a starting point for the consultation, so where I would normally have done physical things, measured their height, their weight, do breathing tests, do feet checks for diabetes, take blood, I actually started the consultation with “I see these are the things that you want to talk about today” and then brought the review round their priorities rather than starting from my own viewpoint.” (5001p)
PROMs as part of routine care	Patient monitoring	Extent to which the intervention encourages patient monitoring	“Yeah definitely, especially if you’ve made a change to their treatment. It’s really nice to have some quantifiable feedback to see how that compares to before they had the treatment if that makes sense.” (5001p)
	Patient experience	Nurses’ perceptions of the patient experience	“All the patients that took part in it, all the feedback that they gave to me verbally, was that it was a good experience. Some of them said it was the best review they’ve ever had, they felt they were listened to…” (5001p)
	Patient management	Nurses’ perceptions of patient management	“From my point of view it was quite positive because I think the problems I was dealing with seemed to be well managed and that sort of came across.” (2001p)

(a) Experience of patients completing PROMs

### Ease of administration

Interviews highlighted that patients did not have any major issues with completing the PROMs, with one respondent reflecting, the questionnaires were “easy to understand and accept.” One interviewee stressed that it was interesting to do and that they “enjoyed the challenge of finding the answers”. Patient free text comments highlighted the experience patients had in terms of completing the PROMs, positive and negative. Typical comments included “most impressed with system” and “thoroughly pleasant and intuitive”. However, while the majority of participants evaluated the experience of completing PROMs as positive, negative feedback was also received in the evaluation questionnaires. One patient described the process as “box ticking”, and another stated, “I don’t think this this was a proper use of NHS manpower.”

### Potential alternative modes of administration (electronic)

There were mixed views on the subject of administration being completed electronically as an alternative, for example on a tablet device or computer, either in advance or in the surgery waiting room. In interviews, some participants were neutral about the method of administration (“don't bother me either way”) but one found the prospect of completing PROMs electronically without assistance unappealing “the personal contact in my view is essential.” One of the free-text comments on the patient evaluation form suggested that “some [of the process] could be undertaken online.”

(b) PROMs feedback

### Ease of interpretation

A majority of patients (83%) found the PROMs summary helpful for the review. On the evaluation forms, a high percentage of patients agreed/strongly agreed that the information was easy to understand (95%). Only 7% of patients indicated they would have liked more detailed information in addition to the PROMs summary they received. This view was broadly supported in the patient interviews, with one interviewee highlighting “the simplicity of it is the benefit really” and another that “you don’t need to put three charts together you have a very clear picture.” However, one person suggested the addition of a “few more clinical bits”, such as blood tests, would enhance the document.

A majority of nurses (89%) found the PROMs summary helpful for the review. The benefit of the simplicity of the PROMs feedback was a recurring theme in the nurse interviews, as illustrated by the following two examples, “I found it very simple to look at” and “it was great. Nice and simple. I like simple…” The layout of the form was well-received (“good and clear”). Interviews also indicated that nurses found the supporting information (which patients did not receive) on interpreting the PROMs scores useful (see Appendix 2 for an example). In one case they said they referred to it “because it was helpful just to understand a little bit more about where the patient had graded themselves.”

### Facilitating awareness and prioritization

Patient interviews indicated that the PROMs summary provided an accurate reflection of health and capabilities, “…it just puts down in black and white how I felt at the time.” One participant stressed that the summary encouraged awareness, relating how they discussed with their husband, “some of the aspects of my condition that I perhaps hadn’t considered before”.

In terms of prioritization, patients indicated that the PROMs summary provided a useful way of prioritizing health issues, as illustrated by both free-text comments on the exit survey (“useful in establishing priorities”) and in interviews (“[it] changed the priority from the outside view to my view”). Similarly, nurse interviews highlighted that the PROMs summary provided a way of prioritizing what was important to patients, “…making sure that we’re not missing the thing the person came into talk about.” Another mentioned that “I used their health priorities on your form as a starting point for the consultation”, instead of the usual clinical focus. This point was also stressed in the evaluation questionnaires, for example, “it was really nice to make the focus of the review the patient and not the tasks. I think we had a more meaningful discussion as a result.”

(c) PROMs as part of routine care

Patient interviews indicated the potential benefits of monitoring PROMs as a part of routine care. One interviewee reflected, “from my point of view it’s either going to highlight a problem or give you a reassurance that you know things are remaining good.” Another person expressed a view that it is a tool “which can be, should be, ought to be used.” The main barrier mentioned, concerning the PROMs intervention forming part of routine care, was about the availability of resources. For example, one patient questioned how useful it would be to their GP surgery, “because they’re all so busy and they’ve got so much else on their plates”.

Interviews with nurses indicated support for including PROMs as part of routine patient care. “some of them said it was the best review they’ve ever had, they felt they were listened to…” Potential benefits in terms of monitoring were highlighted, “if you’ve made a change to their treatment it’s really nice to have some quantifiable feedback to see how that compares to before they had the treatment.” The PROMs focus was viewed positively by another nurse, “From my point of view it was quite positive because I think the problems I was dealing with seemed to be well managed and that sort of came across.” However, one nurse wrote on their evaluation form “not really relevant in this case. They manage their illness well.”

## Discussion

We have developed a theory-informed, PROMs based, nurse-oriented intervention following the MRC framework. After tailoring it to patients with multimorbidity, we have obtained preliminary confirmation of the feasibility and acceptability of the intervention in a pilot study. We were able to successfully train all the nurses and all participating patients were able to complete PROMs, receive personalized feedback ahead of their scheduled review and confirmed that the nurses were aware of their scores. The majority of participating patients reported a positive experience about the process of completing PROMs, finding it intuitive, some enjoyed the challenge of reflecting on their health and finding answers, and no major issues were reported.

### Strengths and limitations

We followed a model proposed in previous literature utilizing PROMs as a means of facilitating person-centred reviews [5, 10], taking into account the views of patients and professionals. In doing so we have incorporated available guidance on multimorbidity, in that the real-time collection and feedback of PROMs constitutes an important way to address the stated aim of being responsive to individual needs, preferences for treatments, health priorities, lifestyle and goals [45]. By aligning our intervention with existing clinical processes (primary care patient reviews) and incorporating adequate training for nurses, we have addressed limitations associated with the past unsuccessful implementation of PROMs in Primary Care [5, 46, 47].

We also need to highlight some important limitations of our study. Firstly, we recruited 68 patients falling short of our goal of recruiting 75 (although we did receive 75 expressions of interest). The main reason for this was that in one small rural practice it was only possible to recruit 8 patients. While we were able to overrecruit from another, larger practice, this has provided us with useful information for the design of the evaluation of a subsequent effectiveness study. Secondly, we opted for a pen and paper version of the tools in consultation with a Primary Care professional. However, in the current environment, particularly in relation to the recent surge in the uptake of online solutions as part of the provision of routine primary care in response to the COVID pandemic, electronic administration should be considered. Finally, this feasibility and acceptability study was based on a small sample size and had a non-randomized design. Our aim was not to elicit evidence on the effectiveness of the proposed intervention, but rather to advance in the pathway to a randomized evaluation. Such a trial has been designed and is expected to be implemented soon.

### Comparison of findings with previous literature

This study makes an original contribution to the literature as it is the first PROMs based intervention that has been specifically developed for people with multimorbidity. It is also novel in that it combines the use of three different types of PROMs: a standardized generic PROM, multiple standardized disease-specific PROMs and an individualized PROM. However, information on its effectiveness is still lacking which is something we aim to address in a future iteration of the intervention. This would add to a growing body of work demonstrating the benefits of PROMs in routine clinical practice. Systematic reviews on the impact of feeding back information to nurses have demonstrated a positive impact on processes of care, particularly on diagnosis, and increasingly positive impact on outcomes of care [11, 48]. A recent realist synthesis has linked feedback to improved patient-clinician communication and indicates that PROMs completion is not a neutral act of information retrieval but can change how patients think about their condition. Further, a Cochrane review is underway and will facilitate an updated synthesis of the available evidence [32].

### Implications for research and practice

A minority of participants highlighted issues which we will aim to rectify in future work. The evaluation questionnaires raised concerns that the study was perceived as a box ticking exercise and not an effective use of NHS “manpower”. This is something we aim to address in developing an electronic version of the intervention including all of the administration, scoring and automated feedback.

The PROMs feedback had a high level of acceptability. A simple, easy to digest summary was seen as a key, although nurses appreciated being able to draw on supporting information if needed, and some patients expressed a preference to be given the option to access more detailed information which is something we will seek to take on board in future development work. A particular strength of the PROMs summary was that it was seen as helping patients prioritize which aspects of their health they wanted to focus on and that these priorities were in turn communicated to nurses by the summary. Both patients and nurses were on the whole keen for PROMs to be incorporated as part of routine clinical care. Patient monitoring was seen as a potential advantage of the intervention, however, a clinician raised doubts concerning the usefulness for one of their patients in that they were already well managed. On the one hand, it can be argued that it may not be possible to know if patients are well managed without formal appraisal of outcomes from the patient perspective. On the other hand, it may suggest that there may be scope in the evaluation of the effectiveness of the intervention (which was beyond the scope of the present study) for examining the differential impact in subgroups of patients defined by the level of their disease control.

This feasibility evaluation does not provide robust evidence for changing clinical practice but is of significant interest for research, as it is the first demonstration of the potential of PROMs feedback in this patient group, and in particular in exploring tailoring feed-back to patients. It also provides evidence for the potential of using standardized and individualized tools complementarily. In the future, we aim to build on this work to develop digitalized versions of the main components of the intervention  with the potential to be administered on a larger scale. This would entail both replacing current administration and feedback with a state of the art multiplatform electronic system as well as developing an online training programme for nurses. Firstly, by developing an efficient and user-friendly electronic system to enable real-time electronic administration, scoring, interpretation and feedback of PROMs to participating patients and healthcare nurses, in a way which coincides with annual reviews, causing minimal disruption for participating patients and healthcare staff. Secondly, by putting together an online training package on the use PROMs in clinical practice, addressing rationale, evidence, and practicalities of everyday use, with a particular focus on the interpretation of scores of standardized measures and prioritization and goal setting of care based on individualized measures. We intend to implement a small scale randomized pilot of the new digitalized intervention, incorporating an assessment of feasibility and acceptability along with a preliminary evaluation of effectiveness, before moving ahead with a larger-scale randomized controlled trial.

### Conclusion

This study has demonstrated that is it feasible to deliver a PROMs based intervention for patients with multiple chronic conditions, incorporating standardized and individualized measures, coinciding with existing Primary Care reviews, in a way that is acceptable to both patients and health nurses.

## Data Availability

The datasets used and/or analysed during the current study are available from the corresponding author on reasonable request.

## Appendix 5: Themes in patient free text comments

**Table:**

Theme	Frequency	Examples
General evaluation about the process (e.g. generally pleased/happy to take part; thought it was comprehensive/efficient; positive researcher impact)	13	“I thought it was excellent” “Most impressed with system” “Thoroughly pleasant and intuitive”
Patient-centred approach (e.g. holistic approach, feeling listened to)	5	“I think it is vital to see patient as a whole person” “I felt listened to and responded to”
Awareness of health issues	2	“Probably made me more aware of my personal frailties” “Useful in establishing priorities”
Suggestions for improving the process (e.g. querying, pointing out limitations/negative aspects)	5	“Some could be undertaken online” “Box ticking” “I don’t this this was a proper use of NHS manpower”

## Appendix 6: Themes in nurse free-text comments

**Table:**

Theme	Frequency	Examples
Patient centeredness	9	“It was nice to make the focus of the review the patient and not the tasks” “Allows holistic approach.”
Facilitated discussion of patient priorities	7	“Prompted discussion of priorities”
Lacking relevance	5	“Not really relevant in this case. They manage their illness well”
Ease of use	2	“Patient needed assistance with answering questionnaire”
Patient awareness of their health	2	“Useful to show that patient is aware how well they have progressed”
Patient management	1	“Changed my approach”
Patient satisfaction (explicitly stated that the patient was satisfied with the review)	1	“Patient very impressed with the study”
General positive feedback	8	“Discussed health priorities with patient & patient found it helpful”
Neutral, generic and otherwise non-applicable comments	11	“This patient lives a normal life with his diseases”

## Abbreviations

Mini-AQLQ: Mini Asthma Quality of Life Questionnaire
CCQ: Clinical COPD questionnaire
COPD: Chronic Obstructive Pulmonary Disease
DHP: Diabetes Health Profile
GP: General Practitioner
MLHFQ: Minnesota Living with Heart Failure Questionnaire
MRC: Medical Research Council
NHS: National Health Service
NICE: The National Institute for Health and Care Excellence
OHS: Oxford Hip Score
OKS: Oxford Knee Score
OSOP: One Sheet of Paper
PHQ-9: Patient Health Questionnaire
PGI: Patient Generated Index
PROM: Patient Reported Outcome Measure
RCP: Royal College of Physicians
